# SARS-CoV-2 RNA detected in blood products from patients with COVID-19 is not associated with infectious virus

**DOI:** 10.12688/wellcomeopenres.16002.2

**Published:** 2020-10-12

**Authors:** Monique I. Andersson, Carolina V. Arancibia-Carcamo, Kathryn Auckland, J. Kenneth Baillie, Eleanor Barnes, Tom Beneke, Sagida Bibi, Tim Brooks, Miles Carroll, Derrick Crook, Kate Dingle, Christina Dold, Louise O. Downs, Laura Dunn, David W. Eyre, Javier Gilbert Jaramillo, Heli Harvala, Sarah Hoosdally, Samreen Ijaz, Tim James, William James, Katie Jeffery, Anita Justice, Paul Klenerman, Julian C. Knight, Michael Knight, Xu Liu, Sheila F. Lumley, Philippa C. Matthews, Anna L. McNaughton, Alexander J. Mentzer, Juthathip Mongkolsapaya, Sarah Oakley, Marta S. Oliveira, Timothy Peto, Rutger J. Ploeg, Jeremy Ratcliff, Melanie J. Robbins, David J. Roberts, Justine Rudkin, Rebecca A. Russell, Gavin Screaton, Malcolm G. Semple, Donal Skelly, Peter Simmonds, Nicole Stoesser, Lance Turtle, Susan Wareing, Maria Zambon

**Affiliations:** 1 Oxford University Hospitals NHS Foundation Trust, John Radcliffe Hospital, Headington, Oxford, OX3 9DU, UK; 2 Translational Gastroenterology Unit, John Radcliffe Hospital, Headington, Oxford, OX3 9DU, UK; 3 Nuffield Department of Medicine, John Radcliffe Hospital, Headington, Oxford, OX3 9DU, UK; 4 NIHR Oxford Biomedical Research Centre (BRC), John Radcliffe Hospital, Headington, Oxford, OX3 9DU, UK; 5 Roslin Institute, The University of Edinburgh, Easter Bush Campus, Midlothian, EH25 9RG, UK; 6 Sir William Dunn School of Pathology, University of Oxford, South Parks Road, Oxford, OX1 3RE, UK; 7 Department of Paediatrics, University of Oxford, John Radcliffe Hospital, Headington, Oxford, OX3 9DU, UK; 8 Porton Down, Public Health England, Manor Farm Road, Porton Down, Salisbury, SP4 0JG, UK; 9 Big Data Institute, Roosevelt Drive, Old Road Campus, Headington, Oxford, OX3 7LF, UK; 10 NHS Blood and Transfusion, 26 Margaret St, Marylebone, London, W1W 8NB, UK; 11 University College London, Gower St, Bloomsbury, London, WC1E 6BT, UK; 12 Public Health England, 61 Colindale Ave, London, NW9 5EQ, UK; 13 NHS Blood and Transplant, John Radcliffe Hospital, Headington, Oxford, OX3 9DU, UK; 14 Nuffield Department of Surgical Sciences, University of Oxford, John Radcliffe Hospital, Headington, Oxford, OX3 9DU, UK; 15 Component Development Laboratory, NHS Blood and Transplant, Cambridge Donor Centre, Cambridge, CB2 0PT, UK; 16 Nuffield Department of Population Health, University Oxford Richard Doll Building, Old Road Campus, Headington, Oxford, OX3 7LF, UK; 17 NIHR Health Protection Research Unit in Emerging and Zoonotic Infections, Faculty of Health and Life Sciences, University of Liverpool, Liverpool, L69 3BX, UK

**Keywords:** COVID-19, SARS-CoV-2, viral load, viraemia, RNA, blood, biomarker, laboratory safety

## Abstract

**Background: **Laboratory diagnosis of SARS-CoV-2 infection (the cause of COVID-19) uses PCR to detect viral RNA (vRNA) in respiratory samples. SARS-CoV-2 RNA has also been detected in other sample types, but there is limited understanding of the clinical or laboratory significance of its detection in blood.

**Methods: **We undertook a systematic literature review to assimilate the evidence for the frequency of vRNA in blood, and to identify associated clinical characteristics. We performed RT-PCR in serum samples from a UK clinical cohort of acute and convalescent COVID-19 cases (n=212), together with convalescent plasma samples collected by NHS Blood and Transplant (NHSBT) (n=462 additional samples). To determine whether PCR-positive blood samples could pose an infection risk, we attempted virus isolation from a subset of RNA-positive samples.

**Results: **We identified 28 relevant studies, reporting SARS-CoV-2 RNA in 0-76% of blood samples; pooled estimate 10% (95%CI 5-18%). Among serum samples from our clinical cohort, 27/212 (12.7%) had SARS-CoV-2 RNA detected by RT-PCR. RNA detection occurred in samples up to day 20 post symptom onset, and was associated with more severe disease (multivariable odds ratio 7.5). Across all samples collected ≥28 days post symptom onset, 0/494 (0%, 95%CI 0-0.7%) had vRNA detected. Among our PCR-positive samples, cycle threshold (ct) values were high (range 33.5-44.8), suggesting low vRNA copy numbers. PCR-positive sera inoculated into cell culture did not produce any cytopathic effect or yield an increase in detectable SARS-CoV-2 RNA. There was a relationship between RT-PCR negativity and the presence of total SARS-CoV-2 antibody (p=0.02).

**Conclusions: **vRNA was detectable at low viral loads in a minority of serum samples collected in acute infection, but was not associated with infectious SARS-CoV-2 (within the limitations of the assays used). This work helps to inform biosafety precautions for handling blood products from patients with current or previous COVID-19.

## Background

Since January 2020, the SARS-CoV-2 virus has caused a global pandemic of COVID-19, challenging hospitals and laboratory services worldwide ^1^. Diagnosis of infection has largely been based on RT-PCR amplification of viral nucleic acid from the upper respiratory tract (nose/throat) swabs ^2^. However, detection of viral RNA (vRNA) has also been reported in blood, serum and plasma from clinical small case series (e.g. 3, 4). The frequency and quantification of SARS-CoV-2 RNA in blood fractions, and the significance of blood as a transmission route remains unknown.

Understanding the clinical contexts within which SARS-CoV-2 RNA can be detected in blood is important to determine the extent to which PCR-positive blood, plasma or serum could have impact as a clinically useful biomarker of disease severity or prognosis. Furthermore, there is an urgent need to consider whether the detection of viral RNA in blood samples reflects the presence of infectious virus, as this has important safety implications for clinicians and laboratory personnel engaged in both routine laboratory testing, as well as SARS-CoV-2-specific pipelines such as serology ^5, 6^.

Different organisations have made varying recommendations for the laboratory handling of samples from patients with suspected or confirmed SARS-CoV-2, but these have had to be developed quickly in the face of little experience or data, and rely on the presence of viral RNA in samples as an imperfect surrogate for live virus. Laboratory protocols seeking to reduce the bioburden of SARS-CoV-2 in clinical samples suggest either chemical inactivation (e.g. with sodium-dodecyl-sulfate, Triton-X100, and/or guanidinium thiocyanate-lysis buffers), alone or in combination with heating protocols that vary from 30°C up to as high as 92°C for 15 minutes ^7^. These approaches add processing time, may require additional laboratory reagents, and are also potentially associated with a loss of sensitivity in any downstream analysis, particularly pertinent for serological assays. Previous reports suggest that heat inactivation may be particularly detrimental to the sensitivity of antibody detection ^8^.

An alternative to chemical or heat inactivation is to undertake all sample handling in a biosafety (containment) level 3 (BSL3) facility, but this is expensive, requires specialist staff training, substantially reduces the number of samples that can be processed, and is completely inaccessible in many settings. There is a lack of consensus about appropriate biosafety precautions, and escalation to BSL3 may be based on concerns about risks associated with viraemic samples even when the risk of aerosol generation is low, and there are no data to suggest a risk of blood-borne transmission to laboratory staff.

Here we assimilate the peer-reviewed literature describing the presence of SARS CoV-2 RNA in human blood, with the aim of providing a pooled dataset to provide improved insights into the causes and correlates of RNA-aemia. We then present our own investigation of the frequency and determinants of vRNA detection in blood using 424 samples collected from acutely infected and convalescent patients infected with SARS-CoV-2. We attempted *in vitro* isolation of the virus from viraemic samples in order to determine whether RNA detection is a marker of infectious virus. Together, these data may help to determine the significance of viral RNA in blood, and can contribute to the development of consistent and evidence-based laboratory protocols.

## Methods

### Terminology and definitions

- **Blood:** we have used the term blood to refer to whole blood, serum or plasma when there is not a clear distinction in existing pre-published data, although we recognise that there may be differences in the sensitivity of viral detection between whole blood and blood fractions.
- **Serum:** in the work undertaken here, we refer specifically to serum, as this blood fraction was consistently used across our experiments.
- **RNA-aemia:** we have used this term to describe the presence of viral RNA, above the technical limits of detection of RT-PCR assays, in blood, serum or plasma. The alternative term, ‘viraemia’, suggests the presence of whole virus in blood. Since we have not demonstrated the presence of replication-competent (infectious) SARS-CoV-2 in the blood compartment, we have elected to use the more conservative description of RNA-aemia (which may or not indicate viraemia).

### Systematic literature review

**Figure 1.**
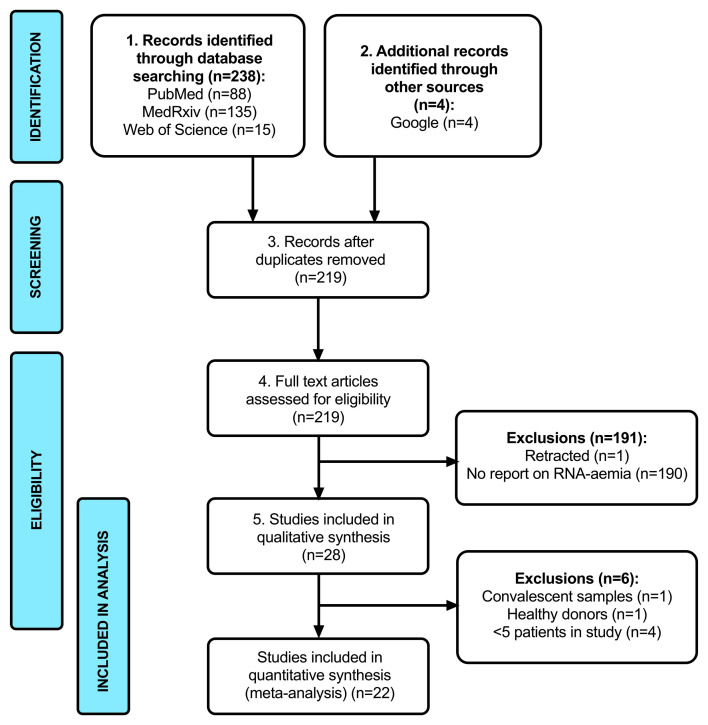
PRISMA flow diagram showing number of abstracts identified through a systematic literature review, rejections (with reasons), and final number of studies included in the analysis.

**Figure 2.**
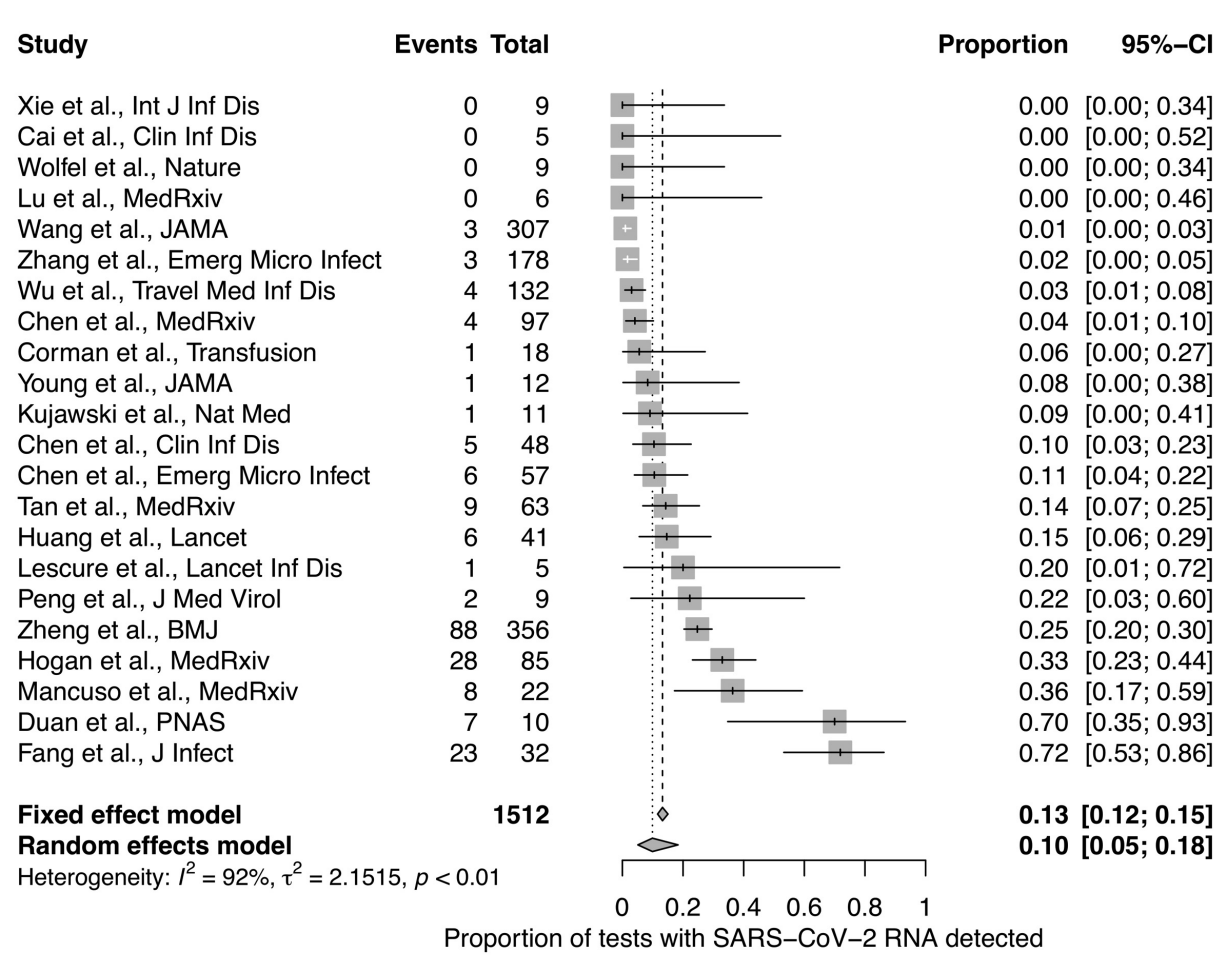
Prevalence of SARS-CoV-2 RNA in serum / plasma / whole blood samples from a systematic literature review. Point prevalence indicated for each study with confidence intervals showing citation and number of samples represented ( Table 1).

We searched PubMed, Web of Science, MedRxiv and Google between 7th-11th May 2020, using the search terms (“SARS-CoV-2” OR “COVID” OR “2019-nCoV” OR “COVID-19” OR "2019 NCOV" or "SARS COV 2" or "2019NCOV" or "2019-nCoV" or "2019 novel coronavirus") AND (“qPCR” OR “RT-PCR” OR “PCR” OR “VIRAL LOAD” OR “RNAaemia” OR “RNAemia” OR “viraemia” OR “viremia” OR “RNA-aemia” OR “RNA-emia”) AND (“BLOOD” OR “PLASMA” OR “SERUM”). We excluded animal studies. We did not make exclusions on the basis of language, but two papers not in English were ruled out because they did not contain details of vRNA detection that we required. Each study was reviewed by at least two independent reviewers. A PRISMA flow chart is presented, showing identification of 28 relevant studies ( Figure 1; Extended Data Table 1 ^9^). We collected information on the prevalence of vRNA detection in blood, serum or plasma, noting whether this attribute was correlated with clinical or laboratory phenotypes of disease, and recording cycle threshold (Ct) values when these were reported. Data were collated in Microsoft Excel v16.31. To allow appraisal of quality and identification of bias, we recorded the number of participants in each study, the location and nature of the study cohort, and (where available) the severity of illness and the timing of sample collection relative to symptoms or PCR-diagnosis. To reduce bias in the meta-analysis, we removed one study each of uninfected (healthy) donors and convalescent individuals, and four studies with <5 participants, taking the final number of studies analysed to 22 ( Figure 2).

**Table 1.** Frequency of SARS-CoV-2 RNA in human blood and blood products based on a systematic literature review. Full metadata are presented in Extended Data File 1, available online ^9^.

Citation	Setting	Frequency and characteristics of SARS-CoV-2 RNA
ACUTE COVID-19 INFECTION		
Wang *et al.*, *JAMA* ^4^	n=205 patients with COVID-19; Hubei and Shandong provinces and Beijing, China	• Blood: 3/307 samples RNA positive collected from 205 patients (0.98%); mean Ct 34-35 • No difference in Ct values between blood, stool, and respiratory samples
Zhang *et al.*, *Emerging* *Microbes & Infections* ^13^	n=178; Wuhan pulmonary hospital, China	• Whole blood: 6/178 (3.4%) PCR positive; Ct 30-32 • Serum: 3/178 (1.7%) PCR positive; Ct 24-33 • None of the patients with viral RNA detected in blood had positive respiratory swabs
Lescure *et al.*, *Lancet* *Inf. Dis.* ^14^	n= 5, hospital patients, France	• Plasma: 1/5 (20%) PCR positive; Ct >35 • Latest positive 12 days after symptom onset. • The patient with vRNA-aemia was the most severely ill.
Duan *et al.*, *PNAS* ^15^	n= 10, severe COVID19 patients, Wuhan, China	• Serum: 7/10 (70%) PCR positive; Ct 34-38
Chen *et al.*, *CID* ^16^	n=48, General Hospital of Central Theater Command, PLA, Wuhan, China	• Serum: 5/48 (10%) PCR positive • RNAaemia only in the critically ill group (but 12 critically ill patients had no RNA-aemia) • RNA-aemia associated with elevated IL-6
Chen *et al.*, *Emerg* *Microbes Infect.* ^17^	n=57, Guangzhou Eighth People’s Hospital, China	• Serum: 6/57 (11%) PCR positive; Ct 32-41 • RNA-aemia associated with severe symptoms
Fang *et al.*, *J. Infect.* ^18^	n=32, Central Hospital of Xiangtan, China	• Blood: 7/8 (88%) PCR positive in ICU patients and 16/24 (67%) in non-ICU patients.
Han *et al.*, *CID* ^19^	n=2, Seoul Metropolitan Government-Seoul National University, Korea	• Mother and 27 day old infant • Plasma: RNA detected in infant up to day 10, mother’s plasma negative
Huang *et al.*, *Lancet* ^20^	n=41, hospitalised patients, Jin Yin-tan Hospital, Wuhan, China	• Plasma: 6/41 (15%) PCR positive • No difference in ICU admissions between patients with and without RNA-aemia.
Yu *et al.*, *CID* ^21^	n=4, Beijing Ditan Hospital, Capital Medical University, Beijing, China	• Blood: 0/4 (0%) PCR positive
Young *et al.*, *JAMA* ^22^	n= 18, hospitalized patients, Singapore	• Blood: 1/12 (8%) PCR positive
Xie *et al.*, *Int J Inf Dis.* ^23^	n=9, Sichuan Provincial People’s Hospital and Sichuan Mianyang 404 Hospital, Chengdu, China	• Blood: 0/9 (0%) PCR positive
Wu *et al.*, *Travel Med Inf * *Dis.* ^24^	n=132, The East Section of Renmin Hospital of Wuhan University, China	• Blood: 4/132 (3.03%) PCR positive
Cai *et al.*, *CID* ^25^	n=5, Childrens’ hospital, Shanghai	• Serum: 0/5 PCR positive within 2-3 days of symptom onset
Zheng *et al.* BMJ ^3^	n= 96 admitted patients Zhejiang province, China	• Serum: 39/96 (41%) overall (6/22 (27%) in mild cases, and 33/74 (45%) in severe case) • No difference in viral load between mild and severe cases • Serum had the lowest viral load compared with stool and respiratory samples.
Wolfel *et al.*, *Nature* ^26^	n=9, hospitalised, Munich, Germany	• Serum: 0/9 (0%) PCR positive
Kujawski *et al.*, *Nature* *Medicine* ^27^	n=11, hospitalised patients, USA	• Serum: 1/11 (9%) PCR positive • Detection of RNA in serum associated with clinical deterioration
Peng L *et al.*, *J Med* *Virology* ^28^	n=9, hospitalised patients, Sun Yat‐sen University, China	• Whole blood: 2/9 PCR positive
Corman VM *et al.*, *Transfusion* ^29^	n=18, range of patients, Germany	• Serum: 1/18 PCR positive, in patient with ARDS needing mechanical ventilation. • SARS-CoV-2 present at 179 copies/ml
Song *et al.*, *MedRxiv* ^30^	n=1, China	• Plasma: 0/1 positive
Lu *et al.*, *MedRxiv* ^31^	n=6, hospitalised patients,. Jiangsu, China.	• Serum: 0/6 positive
Mancuso *et al* *MedRxiv* ^32^	n=22 (10 severe disease, 12 mild disease), Milan, Italy	• Plasma: 6/10 RNA positive in severe group (60%) and 2/12 (1.6%) in the mild group.
Hogan *et al.*, *MedRxiv* ^33^	n=85, California, USA	• Plasma: 28/85 detectable RNA • Median Ct value 37.5 (compared with 27.1 for nasopharyngeal aspirate) • Those with RNA-aemia were older and more likely to go to ICU and need mechanical ventilation • All deaths occurred in those with RNA-aemia
Tan *et al.*, *MedRxiv* ^34^	n=67, Chongqing, China	• 9/63 (14%) positive for RNA
Chen *et al.*, *MedRxiv* ^35^	n-97, Zhuhai, China	• Whole blood: 4/97 • All 4 patients with RNA-aemia had the lowest oxygenation
Bouadma *et al.*, *MedRxiv* ^36^	n=1, Paris, France	• Blood: 1/1 RNA detected • Patient developed multi-organ failure and died
CONVALESCENT PATIENTS (>28 days)		
Ling *et al* *Chinese* *Med J* ^37^	n=14, convalescent patients	• Serum: 0/14 (0%)
HEALTHY DONORS		
Chang *et al.*, *Emerging* *Infectious Diseases* ^38^	n= 7425 Healthy blood donors, Wuhan Blood Center, China. Collected Jan-March 2020, peak epidemic.	• Prospective testing of 1,656 platelet donations and 774 whole blood donations: 1/2430 RNA positive (0.04%) • Retrospective testing of whole blood donations: 3/4995 RNA positive (0.1%)

### Cohorts and sample selection

The origin of serum samples, together with supporting metadata, are available in Underlying Data File 1 ^9^. We collected 212 serum samples through the microbiology department at Oxford University Hospitals NHS Foundation Trust (OUH NHSFT), comprising adults with SARS-CoV-2 infection confirmed by a clinical diagnostic microbiology laboratory using RT-PCR on a respiratory swab. These were derived from three groups as follows:

(i) ****Hospital in-patients****, n=139 samples from 94 participants; these were collected from individuals admitted to OUH NHSFT, a tertiary referral centre in the South East of England, for treatment of COVID-19. Samples were collected between 1–5 days following admission to hospital or intensive care (whichever came later), a median of 8 days following symptom onset (range 1-37 days).
(ii) ****Convalescent healthcare workers****, n=41 samples from 41 participants; these were collected from healthcare workers from OUH NHSFT, following a period of ≥ 7 days absence from work following a diagnosis of COVID-19, a median of 12 days following symptom onset (range 7–17 days).
(iii) ****Convalescent patients****, n=32 samples from 32 participants; these were collected from patients presenting to OUH NHS FT followed up in the community, a median of 42 days following onset of COVID-19 symptoms (range 31–62 days).

Additional samples were collected through NHS Blood and Transplant (NHSBT), as follows:

(vi) ****Convalescent plasma donors****, n=142 samples from 142 volunteer plasma donors, ≥28 days from recovery of symptoms. Retrospective confirmation of COVID-19 infection was based on a EuroImmun IgG antibody titre (threshold ratio ≥1.1, based on the manufacturer’s instructions, Underlying data table 1).
(v) ****Healthy pre-pandemic controls****, n=5 samples from 5 independent healthy volunteer donors, collected prior to December 2019.

In groups (i)–(iii) >1 sample was obtained from 45 individuals, so our clinical dataset overall represents 167 unique individuals with COVID-19. Among these 167 individuals, we classified severity of illness as asymptomatic, mild, severe, or critical based on standard WHO criteria ^10^. All serum samples were frozen in 0.5ml aliquots at -20℃.

### RT-PCR on serum samples

Following nucleic acid extraction, we used reverse transcription (RT)-PCR to amplify SARS-CoV targets from serum samples. PCR primer sequences are available in a supporting on-line file set ^9^. Due to different pathways for patient recruitment and sample processing, PCR protocols varied by cohort, as follows:

- Samples from acute hospital admissions and convalescent health care workers were processed by the OUH NHSFT clinical microbiology laboratory (UKAS accredited to ISO 15189:2012), using a Quiagen Symphony Rotorgene protocol with an RNA-dependent RNA polymerase (RdRP) gene target, validated by Public Health England (PHE) for use on respiratory samples ^11, 12^. We used the QIAgen OBL complex 200 extraction method, using QIAsymphony DSP virus/pathogen mini kit (Qiagen 937036), adding 200µl of serum sample to 430µl QIAsymphony complex off-board lysis buffer (Internal Control MS2 RNA, Sigma-Aldrich 10165948001 (0.5µl); Molecular grade water Fisher Scientific 10245203 (1.5µl); RNA carrier, Qiagen 1017647 (9µl); AVE, Qiagen (109µl); PK, Qiagen 19133 (20µl); ATL, Qiagen 157054504 (100µl); ACL, Qiagen 160030311 (190µl)) and eluting into 60µl. Cycling conditions were 55°C for 10 minutes; 94°C for 3 minutes; 45 cycles of 94°C 15 seconds; 58°C for 30 seconds. A report of Ct values, including positive control on each run, was generated by Rotor-Gene Q series software 2.3.1.
- For convalescent OUH NHSFT patients, a nested PCR was undertaken using newly developed PCR primers at the Medawar Building for Pathogen Research, Oxford, targeting the RNA-dependent RNA polymerase (RdRp) gene of SARS-CoV-2 ^39^. For the first round amplification, we generated a 25 µL reaction mix (5 µL RNA extract; 12.5 µL 2X Quantitect Probe RT-PCR Master Mix (Qiagen 204343); 0.5 µL RT mix from the kit; 5 µL 5X 1st-round primer mix (IDT); 2 µL PCR-grade water). PCR conditions: 50°C for 30 minutes; 95°C for 15 minutes; 40 cycles of 95°C for 15 seconds; 55°C for 30 seconds; 68°C for 1 minute; 68°C for 5 minutes. For the second round amplification, we generated a 25 µL reaction mix (1 µL of the first round product; 5 µL 5X GoTaq Green Master Mix (Promega M7841); 0.125 µL 5u/µL GoTaq G2 polymerase (Promega M7841); 2 µL 2.5 mM dNTP mix (Stratech NU-1020S-JEN-200ul); 5 µL 2nd-round 5X primer mix (IDT); 11.875 µL PCR-grade water). PCR conditions: 95°C for 5 minutes, 40 cycles of 95°C for 30 s, 55°C for 30 seconds, 72°C for 1 minute, final extension of 72°C for 5 minutes. The presence of SARS-CoV-2 RNA was confirmed through visualization of the PCR product via UV-Vis agarose gel electrophoresis. The assay demonstrated a 95% detection rate for 13 RNA copies of SARS-CoV-2 RNA transcript spanning the amplified region ^39^.
- Convalescent samples collected through NHSBT were analysed by Public Health England (Colindale), targeting either RdRp or a conserved region of the open reading frame (ORF1ab) gene of SARS CoV-2, together with detection of an assay internal control to monitor the extraction and RT-PCR processes. Reverse transcription and PCR amplification was performed on an Applied Biosystems 7500 FAST system. Samples were aliquoted into lysis buffer containing an exogenously added internal control (soil-borne cereal mosaic virus (SBCMV) RNA transcripts ^40^), prior to purification of nucleic acid. Total nucleic acid was extracted from samples using the Biomérieux NucliSENS easyMAG or eMAG system. Extracted nucleic acid was analysed by either an RT-PCR assay targeting the RDRP gene of SARS -2 CoV as previously described with minor modifications ^12^ or a conserved region of the open reading frame (ORF1ab) gene. This assay uses the primers and probe sequences made public by CDC China ^41^, together with detection of SBCMV IC. ORF1ab primers (100μM) were obtained from Metabion, Planegg, Germany, and ORF1ab probe (10μM) from Tib-Molbiol, Berlin, Germany. A 25μL RT-PCR reaction contained: 5μL of RNA; 12.5μL of 2x reaction buffer; 0.4μL MgSO _4 _(50mM) provided with the Superscript III one step RT-PCR system with Platinum Taq Polymerase (Cat.11732088; Invitrogen, Darmstadt, Germany); 0.15μL ORF1ab-F; 0.2μL ORF1ab-R; 0.125μL SBCMV-F; 0.5μL SBCMV-R; 0.5μL ORF1ab-P; 0.125μL SBCMV-P; 1μL of reverse transcriptase/Taq mixture from the kit; 4.5μL molecular grade water. Thermal cycling was performed on an Applied Biosystems 7500 FAST system (Applied Biosystems, Thermo Fisher Scientific, Hemel Hempstead, England), as follows: 55°C for 10 minutes, 94°C for 3 minutes, 45 cycles of 94°C for 15s, 58°C for 30 seconds.

High Ct values (>37.0) are often viewed as being non-specific in clinical diagnostic laboratories depending on the clinical situation. However, for research purposes we collected and reported all Ct values.

### Viral culture system

For viral culture, we used 20 serum samples, designated VC01-20 (identified in Underlying Data File 1 ^9^). VC01-16 comprised acute and convalescent samples that were RT-PCR positive, selected at random from our sample bank, representing samples from 12 individual patients (four individuals were represented at two timepoints), collected at 3-20 days following onset of symptoms. VC17-20 were pre-pandemic control samples. One further sample collected from a pre-pandemic NHSBT serum donation was used as media (VC21).

Samples VC01-20 were provided blinded for viral culture experiments. 50 μL aliquots of samples VC1-VC20 were separately added to 2.4 × 10 ^5^ Vero E6 cells (Cell Bank, Sir William Dunn School of Pathology, University of Oxford) in 24 well plates. Cells were propagated in Dulbecco's Modified Eagle Medium (DMEM) supplemented with 10% foetal bovine serum (FBS). Virus growth assays were done in DMEM supplemented with 1% FBS, glutamine and penicillin/streptomycin, according to published methods ^42^. In parallel, wells of the same number of cells were cultured in triplicate without virus challenge but with 50 μL control serum (VC21), or in duplicate with a stock of Victoria/01/2020 SARS-CoV-2 passage 4 (Oxford) at calculated ten-fold serial dilutions per well of 78, 7.8, 0.78 and 0.078 plaque forming units (pfu) in 50 μL of control serum (VC21).

Wells were observed daily for cytopathic effects (CPE), using images to record all cultures on days 3 and 7 (electronically archived using LabArchives Research Notebook). We took 50 μL samples for vRNA extraction on day 3 post-challenge. Where residual sample volumes permitted, 50 μL aliquots of the respective serum were processed in parallel. In addition, 1 × 10 ^8^ vRNA copies produced by *in vitro* transcription and quantified by droplet digital PCR were spiked into two equivalent control media samples, and processed in parallel, to provide quantification and estimate the loss of vRNA during extraction. All samples were processed for vRNA using QIAamp Viral RNA Mini kits according to the manufacturer’s instructions. RNA extracts were analysed by qRT-PCR, and vRNA copy number was interpolated from the standard curve of Ct value by known copy number. On day 4, 50 μL aliquots of supernatants from cells challenged with VC01-20 were “blind passaged” to fresh cells, and the remaining supernatants were harvested and stored separately at -80C for future analysis. After a further 3 days, we recorded CPE, if any, for second passage cultures.

***RT-PCR of culture supernatant***. To determine whether there had been productive infection of cells *in vitro*, we took aliquots of culture supernatant, including positive and negative controls, and serum samples for qRT-PCR analysis using CDC NP1, CDC NP2 and HKU ORF1b diagnostic panels.

For the CDC NP1 and NP2 assay, we used an N-gene digital droplet quantified in vitro transcribed RNA standard (GenExpress, Germany). For the HKU ORF1b assay, we used qRT-PCR quantified RNA extracts of Victoria/01/2020 SARS-CoV-2 passage 4 (Oxford) as RNA standard, diluted to 10 ^7^ copies/reaction and preparing a serial dilution. We used Luna Universal Probe One-step qRT-PCR kit (New England Biolabs, USA) for all reactions following the manufacturer’s instructions, with either 5 µL or 2.5 µL RNA sample in a 20 µL or 10 µL reaction (see Table in Extended Data File 2 ^9^). Primer and probe concentrations in reactions were as specified by guidelines from the CDC and University of Hong Kong for their respective assays. PCR cycle conditions were: 10 minutes at 55°C, 1 minute at 95°C, followed by 45 cycles of 5 seconds at 95°C and 30 seconds 55°C. We manually adjusted the threshold for all runs to 0.2 and qRT-PCR efficiency was calculated for quality control. We used slopes from RNA standard curves to interpolate vRNA copy numbers in samples. Samples were analysed in six qRT-PCR runs in total. Each qRT-PCR run contained a freshly prepared RNA standard dilution with a quantitative logarithmic range from 10 ^7^ to 10 ^1^ vRNA copies/reaction to calculate vRNA copy numbers across all samples.

### Serological testing of serum samples for antibody to SARS-CoV-2

For a subset of clinical samples collected in Oxford (n=160), we determined an antibody titre using the Siemens SARS-CoV-2 Total (COV2T), Atellica Solution immunoassay analyser, at Public Health England Porton Down. This assay measures total antibody to Spike protein S1 Receptor Binding Domain (RBD). In a recent evaluation of performance, undertaken collaboratively by Public Health England and Oxford University, this assay had a sensitivity of 98.1% (95% CI 96.6, 99.1) and specificity 99.9% (95% CI 99.4, 100), making it the best performing of the commercially available platforms assessed ^43^. The assay was undertaken at Public Health England, Porton Down, according to the manufacturer’s instructions, with a threshold of ≥1.0 standard units for calling a positive test.

### Ethics

Acute hospital in-patients were recruited into the Sepsis Immunomics study (Ref: 19/SC/0296). Convalescent healthcare workers with hospital encounters (n=38) and convalescent patients (n=32) provided informed consent for recruitment into the ISARIC WHO Clinical Characterisation Protocol UK (ISARIC WHO CCP-UK), with ethics approval by the South Central (Oxford C) Research Ethics Committee in England (Ref: 13/SC/0149), and Scotland A Research Ethics Committee in Scotland (Ref: 20/SS/0028). Additional convalescent healthcare workers were recruited by the Oxford GI Biobank, n=3 (approval by Yorkshire and The Humber - Sheffield Research Ethics Committee, ref. 16/YH/0247). Healthy pre-pandemic control samples were used under NHSBT ethics, providing donor consent for their anonymised samples to be used in research.

### Statistical analysis

Anonymised data were stored using Microsoft Excel. We analysed and presented data using GraphPad Prism v.8.3.1. Statistical analyses were undertaken using R 3.6.2. Binomial confidence intervals are presented for proportions. Univariable and multivariable logistic regression models were used to determine associations between detectable vRNA and time since symptom onset, disease severity and patient sex and age, accounting for any non-linear effects of continuous factors using natural cubic splines. Meta-analysis was undertaken using the meta package for R, version 4.12.

## Results

### Literature review to determine the frequency and clinical associations of RNA-aemia

We identified 28 relevant studies ( Table 1; Table S1), among which 22 contained metadata suitable for meta-analysis ( Figure 1). Point estimates for the frequency of vRNA detection are presented for each study representing ≥5 individuals, together with 95% confidence intervals ( Figure 2; Table S1). We observed considerable heterogeneity in the range of estimates for vRNA-aemia, from 0% in several studies ^21, 23, 25, 26, 37^, up to 76% in a report of patients in a critical care setting ^18^. Pooling the data from these reports, the point estimate for the prevalence of vRNA in blood products in the 28 days following symptomatic infection is 10% (95%CI 5-18%, random effects model).

Viral RNA-aemia was reported in association with more severe disease in some studies, including a higher risk of admission to critical care settings, and increased incidence of acute clinical deterioration ^14, 16, 17, 27^. One study reported lower RNA levels in serum compared to other sample sites ^3^, whilst another found RNA levels in blood to be no different to that of other sample types ^4^. In a small number of reports that included specific Ct values, these were typically high, although studies used variable PCR targets and different thresholds for reporting positivity (details of methods and reported Ct values are available in Table S2).

We excluded two studies from the meta-analysis because they focused on cohorts with different characteristics from all other sample sets. One of these reported PCR results from samples taken at timepoints beyond 28 days, among which none contained vRNA ^37^. The other investigated vRNA-aemia in healthy blood donors in Wuhan, China at the time of the peak of the local epidemic in the first three months of 2020, finding vRNA in six samples from among >7000 screened ^38^.

### Frequency and timing of SARS-CoV-2 RNA-aemia in a local cohort

**Figure 3.**
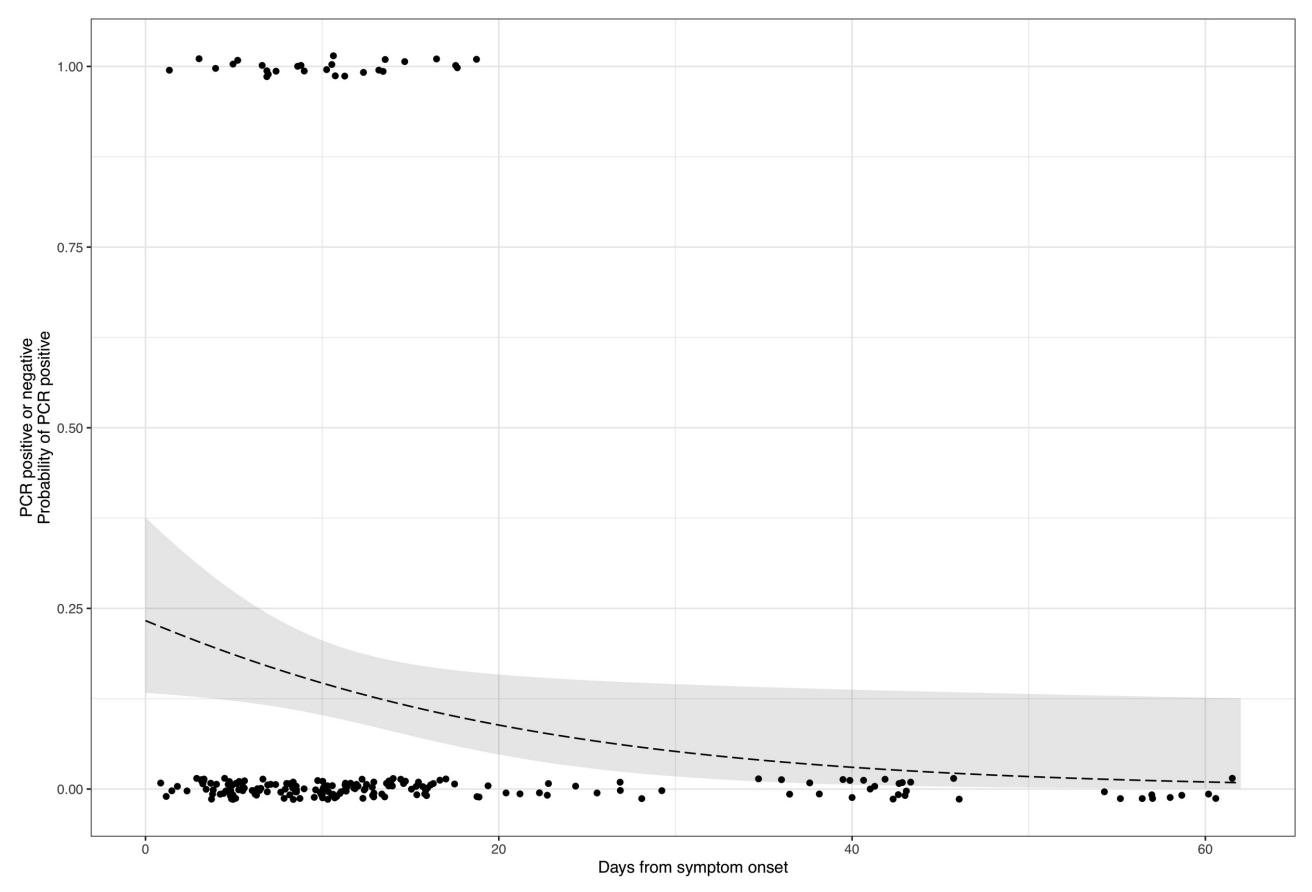
Relationship between RNA-aemia and days from COVID-19 symptom onset. Data shown for 212 samples collected from acute and convalescent adults from the Oxford University Hospitals cohort. Positive and negative results are shown plotted at 1 and 0 on the y-axis respectively, with jitter applied to show all points. The line shows the univariable predicted probability of RNA detection over time (95% CI: shaded).

Our local clinical sample set included n=212 samples from 167 patients (median age 57 years, IQR 46-76), 89 male (53%). In 163 patients for whom clinical data were available, disease was classified as asymptomatic (n=1, 0.6%), mild (n=81, 50.0%), severe (n=37, 22.7%), or critical (n=44, 27.0%). In this sample set, collected at a median of 11 days post symptom onset (IQR 7-17 days), 27/212 were PCR positive for vRNA (12.7%, 95%CI 8.6-18.0%). Deduplicating this to represent 167 unique individuals, 20 (12.0%, 95%CI 7.5-17.9%) had RT-PCR positive serum at any time point tested. Considering all 212 samples in a multivariable analysis, critical disease severity was associated with increased vRNA-aemia, comparing mild and asymptomatic cases to severe (OR 2.3, 95%CI 0.5-12.6, p=0.29) and critical cases (OR 7.5, 95%CI 2.0-37.3, p=0.006) ( Table 2). Within this dataset there was moderate statistical evidence of a trend towards decreased odds of vRNA-aemia over time (OR, per day, 0.95, 95%CI 0.89-1.00, p=0.12) ( Table 2; Figure 3).

**Table 2.** Odds ratios (OR) for associations between RNA-aemia and other patient characteristics, among 212 adults with confirmed COVID-19 infection recruited at Oxford University Hospitals NHS Foundation Trust.

Clinical attribute	Multivariable OR	95% CI		P value
Age, per 10 years	0.97	0.74	1.29	0.85
Sex, Female	1.00	*(reference)*		
Sex, Male	1.54	0.61	4.10	0.37
Severity, Mild or Asymptomatic	1.00	*(reference)*		
Severity, Severe	2.31	0.51	12.64	0.29
Severity, Critical	7.46	2.02	37.31	0.006
Time from symptom onset, per day	0.95	0.89	1.00	0.12

Pooling our hospital data with results from the NHSBT convalescent cohort, vRNA was detected in 23/131 (17.6%, 95%CI 11.5-25.2) samples collected up to day 13, 4/40 samples from between day 14–27 (10.0%, 95%CI 2.8-23.7%), and 0/494 samples at ≥28 days (0%, 95%CI 0.0-0.7%) ( Figure 2B). Day 20 was the latest time point at which any PCR positive sample was collected.

Ct values for all of our 27 PCR-positive sera were high (median 40.9, range 33.6–44.8). Using the more stringent Ct threshold of 37 that may be applied by clinical laboratories to report a positive result, only 7/25 fell below this cut off, reducing our overall positive rate to 7/212 among the local clinical cohort (3.3%, 95%CI 1.3-6.6%) or 7/674 across our entire sample set (1.04%, 95%CI 0.42-2.13%).

### Cytopathic effects arise in cell cultures inoculated with reference viral stock, but not in samples from COVID-19 patients or pre-pandemic controls

**Figure 4.**
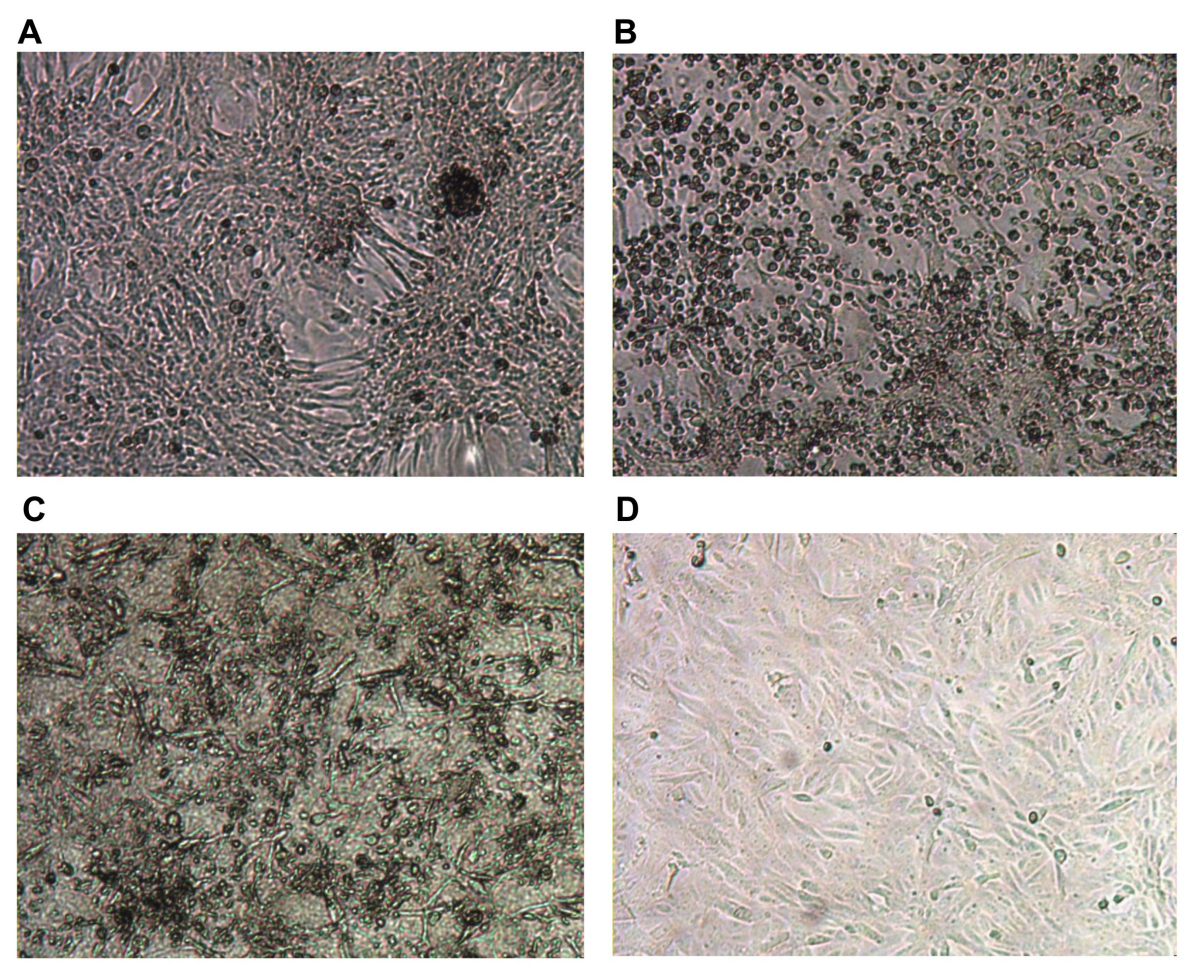
Typical images from cell culture in an *in vitro* system for SARS-CoV-2 culture. Top row shows controls: ( **A**) Negative control Vero E6 cells in media; ( **B**) Cytopathic effect (CPE) in Vero E6 cells spiked with Victoria/01/2020 SARS-CoV-2; Bottom row shows Vero E6 cells inoculated with 1/10 dilution of serum sample from sample VC12 (patient ID UKCOV040), that tested positive for SARS-CoV-2 RNA by RT-PCR; ( **C**) Aberrant cellular effects at day 4 in a culture inoculated with VC12 at day 0; ( **D**) Normal appearance of cells at day 7 inoculated with 1/10 dilution of the culture supernatant of the VC12-challenged culture, illustrated in ( **C**). Raw unedited microscope images can be accessed individually on line ^9^.

Healthy uninfected control cell cultures of Vero E6 cells were established ( Figure 4A). We observed substantial cytopathic effects (CPE) in all samples inoculated with reference virus, characterised by cell rounding up and detaching ( Figure 4B). CPE of this type was observed in wells challenged with 78 and 7.8 pfu, and moderate but typical CPE was observed in one well challenged with calculated 0.78 pfu reference virus. Cells exposed to a 1/10 dilution of control plasma did not show typical viral CPE. However in contrast to the CPE seen with reference virus, these control samples, the VC01-20 test cultures, and the culture inoculated with 0.078 pfu, instead showed variable cellular abnormalities and noticeable gel-formation in most samples ( Figure 4C). Second passage cultures were undertaken in all cases, and none showed evident cytopathic effects at day 7 ( Figure 4D). This approach is limited to the sample volume of 50 μL but we have demonstrated a single pfu at this volume reliably. Our detection threshold is <20 pfu/mL plasma, suggesting that plasma samples contain <0.1 infectious unit per 50 μL.

### RT-PCR of culture supernatant

To determine whether there had been productive infection of cells *in vitro*, we took aliquots of culture supernatant for RT-PCR. From the positive control cultures, any culture receiving ≥1 infectious unit of virus (78 and 7.8 pfu in 50 μL of control serum) on day 0 produced ≥1 × 10 ^5^ copies of viral RNA (vRNA) per sample by day 3, detected by all three (CDC NP1, CDC NP2 and HKU ORF1b) primer/probe sets. All diagnostic panels also detected low levels of vRNA in the culture inoculated with a calculated dose of 0.078 pfu. These vRNA traces are likely to reflect fragments and RNA debris from the cells in which the virus was grown.

No serum sample, and no serum-inoculated cultures had >100 vRNA copies by day 3 based on the CDC, NP1 and NP2 assays. Marginal vRNA was detected in 10 serum samples, but none of these showed a rising titre by day 3 and none had vRNA levels within the reliably quantifiable range. The highest were in the range found in the sub-infectious dose positive control cultures (Extended Data File 2 ^9^). In contrast, no vRNA copies could be detected in serum-inoculated cultures tested at day 3 using the HKU ORF1b primer/probe set, while for the majority of original sera samples the HKU ORF1b assay gave similar results to the CDC, NP1 and NP2 diagnostic panel. The only exception from this was VC15, where marginal vRNA was detected in both serum and serum-inoculated cultures. These results suggest that no rising titre or no vRNA can be detected in serum-inoculated cultures. The comparison between the CDC and HKU diagnostic panels highlights interesting differences for detection of SARS-CoV-2 virus and should be explored further.

### Relationship between serum SARS-COV-2 RT-PCR and antibody titre

**Figure 5.**
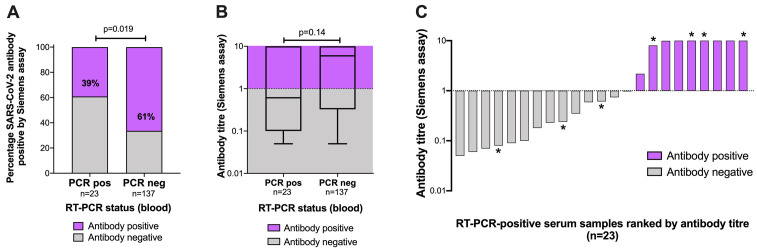
Relationship between serum SARS-COV-2 RT-PCR and total antibody titre, determined by Siemens SARS-CoV-2 Atellica assay. ( **A**) Proportion of samples testing antibody positive according to RT-PCR status of serum sample; p-value by Fisher’s Exact Test; ( **B**) Distribution of IgG titres in samples according to RT-PCR status of serum sample; boxes show median/IQR and whiskers show range; p-value by Mann-Whitney U test; ( **C**) Antibody titres in 23 serum samples testing RT-PCR positive for SARS-CoV-2 RNA, presented in rank order. The bars marked with an asterisk indicate the samples for which *in vitro* culture was attempted. In all panels, antibody positivity is reported according to the threshold set by the assay manufacturer.

We derived SARS-CoV-2 antibody titres for 160 clinical samples using a commercial immunoassay (Siemens); raw data are available in Underlying Data File 1. The antibody test was positive in 100/160 (62.5%) of serum samples tested, and the rate of positivity was higher in PCR-negative compared to PCR-positive samples (p=0.019; Figure 5A). However, there was no quantitative difference in antibody titres according to the PCR-status of the sample (p=0.14; Figure 5B). Among 23 PCR-positive serum samples, antibody titres varied across the dynamic range of the Siemens assay; in this small sample set, the absence of CPE *in vitro* was not dependent on the detection of total antibody ( Figure 5C). 

## Discussion

### Impact of results

Recognition that SARS-CoV-2 RNA may be detected and quantified in blood highlights its potential provenance as a biomarker, but also raises concerns about safety for personnel handling samples in clinical and research environments. Protocols to underpin the safe handling of blood samples need to consider the best evidence for routes and risks of transmission in order to mandate safe laboratory practice, being informed by the nature of the samples and the specific task being undertaken (including any risk of aerosol generation), while also maintaining optimum cost effective workflow of clinical samples. Local risk assessments may currently result in disparate protocols being established by different organisations, but risk assessments should be proportionate, and - as far as possible - unified, and evidence-based. Developing new data to support laboratory practice is an important foundation for standardising practical guidelines.

Based on a systematic review of the literature, together with our own data, we estimate that SARS-CoV-2 RNA may be present at low copy numbers in ~10% of blood samples obtained from individuals with COVID-19 prior to day 28, most of which arise at earlier timepoints and in the setting of more severe disease. Despite being PCR-positive for vRNA, none of our clinical samples exceeded the threshold for viral infectivity.

### Relationship between viral load and disease phenotype

The Ct values reported in the literature and in our local samples are high, reflecting low copy numbers and suggesting that assays may be detecting genomic fragments rather than replication-competent virus in blood. However, it is also possible that intact virions are present, but that these are immune-complexed or otherwise neutralised, accounting for the lack of CPE in our culture system.

A previous study reported a decline in RNA-aemia in severe cases from 45% at the time of admission to 11% by week 4, and in mild cases from 27% to 0% over the same time period, although these differences did not reach statistical significance ^3^. Viral load (measured by qRT-PCR) in respiratory samples has been correlated with disease severity ^3, 44, 45^. Detection of vRNA in blood therefore may be more common in severe/critical disease as a result of higher viral loads overall, or specifically relating to a high burden of infection in the lungs leading to spill-over into the circulation, or reflecting the destruction of infected cells in the respiratory epithelium. Multisystem end-organ disease caused by SARS-CoV-2 could reflect systemic viral dissemination by blood or lymphatics (potentially with direct infection of lymphocytes), or may arise as a consequence of a sepsis syndrome triggered primarily by localised pulmonary infection ^46^. Given the high Ct values for vRNA in blood, the identification of virus in the vascular compartment currently remains non-specific; further work is needed to understand its origins and significance, and to determine whether vRNA in the blood is innocuous or could contribute to immune dysfunction and the systemic inflammatory process.

Further work is needed to determine the bioburden and clinical significance of SARS-CoV-2 in other tissue types, for example in faeces ^47, 48^. Different clinical and laboratory infection control practices should be considered for specific sample types, ideally based on an understanding of the frequency and duration of carriage and assessment of whether infectious virus can be detected.

### Relationship between antibody detection and RNA-aemia

The relationship between RNA-aemia and antibody status may be a causal one, in which neutralising antibodies directly reduce viral titres in blood. However, this association may also be a feature of the time-course of evolving infection, as samples collected early in infection are both more likely to contain a ‘spill over’ of virus from the respiratory tract into blood, and to be seronegative, reflecting a ‘window period’ before seroconversion ^6^. We have not tested neutralising activity on this current sample set, but a number of studies now show a close association between IgG titres and neutralisation, which confirms the biological activity of the antibodies *in vitro* ^49, 50^, suggesting a causal association is entirely plausible. It should also be noted that commercially available platforms for antibody testing are validated on the basis of reporting a binary read-out, based on positive/negative thresholds set by the manufacturer, but neutralising antibodies may be present even below this threshold which could explain the lack of CPE even in samples for which the Siemens assay was reported as negative.

### Caveats and limitations

Datasets reported in the literature represent mostly a small number of carefully selected patients, typically in the acute hospital setting and therefore biased towards inclusion of more unwell patients meeting WHO criteria for severe or critical disease. Recognising that the field that is currently moving at pace, we elected to include papers from the pre-print server MedRxiv, for which peer review has not been undertaken. As a result, not all material included has undergone this quality assurance step. Published reports frequently do not include timing of sample collection relative to diagnostic respiratory samples and/or symptom onset, samples from individuals with trivial or absent symptoms are not well represented in existing studies, and there are insufficient data to distinguish between frequency or quantification of vRNA present in whole blood, versus serum or plasma.

Due to the logistics of rapid recruitment of different patient groups through different pathways, RT-PCR methods varied by cohort, potentially introducing some variation in the sensitivity of detection. In our clinical samples, we adopted an inclusive approach to reporting detection of vRNA, by including samples with Ct values above those which would normally be called positive by a clinical diagnostic facility. This may lead to an over-estimation of the true prevalence of RNA-aemia in this sample group. Many previous publications do not report Ct values and direct comparisons between datasets are therefore difficult.

The absence of CPE and amplification of vRNA must be considered within the constraints of the low sample volume (50 μL in each assay), and the limits of detection within the assays used. We tested serum samples after they had been subjected to a freeze/thaw cycle, which could also have potential influence on retrieval of infectious virus. However, as samples were frozen in accordance with standard laboratory operating protocols within a few hours of collection, we anticipate this would have a limited impact on viral replication capacity, as has been demonstrated previously for other viruses ^51– 53^.

## Conclusions

Our data confirm that blood from COVID-19 patients may contain detectable RNA, but this arises in a minority of samples and is typically in low copy numbers, often outside the threshold that would be reported as positive in a clinical diagnostic laboratory. Based on evaluation of a small sample set, we have found no evidence to suggest that blood samples containing RNA could yield replication competent virus, suggesting a negligible risk of transmission of SARS-CoV-2 to healthcare workers and laboratory staff from handling such material. However, laboratory practice should be informed by guidance from Public Health England ^54^, CDC ^55^ and WHO ^56^; individual risk assessment is important to account for the nature of the material being handled and the process being undertaken. Universal precautions and routine safety procedures should be carefully observed, not only to protect from SARS-CoV-2 infection but also to provide protection from other potential pathogens. Further data are needed to determine the extent to which serum PCR positivity for vRNA is useful as a diagnostic or prognostic marker in patients with COVID-19 infection.

## Data availability

### Underlying data

Figshare: SARS-CoV-2 RNA in blood. https://doi.org/10.6084/m9.figshare.12278249.v8 ^9^.

This project contains the following underlying data:

- Underlying Data File 1.xlsx (Metadata table for serum samples from adults with confirmed SARS-CoV-2 infection, based on RT-PCR nose/throat swab and/or EuroImmun antibody titre)
  Sheet 1: samples obtained through patients recruited into a UK clinical cohort at Oxford University Hospitals NHS Foundation Trust (n=212 samples from 167 unique individuals). Cells highlighted in blue show follow-up samples collected from the same individual at different time points. Cells highlighted in orange show serum PCR positives. All individuals had a diagnosis based on an RT-PCR throat swab positive for SARS-CoV-2. Sheet 2: samples obtained from convalescent donors a minimum of 28 days post resolution of symptoms, via NHS Blood and Transplant, NHSBT (n=142 samples from 142 individuals).
- Fig 4A 20200501_cc1.jpg (raw unedited microscope images for Figure 4A)
- Fig 4B 20200501_1in100_2.jpg (raw unedited microscope images for Figure 4B)
- Fig 4C 20200501_vc12.jpg (raw unedited microscope images for Figure 4C)
- Fig 4D 20200504_vc12.jpg (raw unedited microscope images for Figure 4D)

### Extended data

Figshare: SARS-CoV-2 RNA in blood. https://doi.org/10.6084/m9.figshare.12278249.v8 ^9^.

This project contains the following extended data:

- Extended Data File 1.xlsx (Metadata table providing data for prevalence of SARS-CoV-2 RNA in blood and blood products based on a systematic literature review. Details of 28 citations are presented, and the 22 studies included in quantitative meta-analysis are indicated)
- Extended Data File 2.pdf (qRT-PCR quantification of vRNA from sera and viral culture assays. Calculation of vRNA copy numbers, and qRT-PCR results in figure and table format.)
- RT-PCR Primer sequences.xlsx (Primer sequences)

### Reporting guidelines

Figshare: PRISMA and STROBE checklists for ‘SARS-CoV-2 RNA detected in blood products from patients with COVID-19 is not associated with infectious virus’ https://doi.org/10.6084/m9.figshare.12278249.v8 ^9^.

## References

[1] World Health Organisation Coronavirus disease (COVID-19) Situation Dashboard. who.int. [cited 31 Mar 2020]. Reference Source

[2] Tang YW Schmitz JE Persing DH: The Laboratory Diagnosis of COVID-19 Infection: Current Issues and Challenges. *J Clin Microbiol.* 2020;58(6):e00512–20. 10.1128/JCM.00512-20 32245835 PMC7269383

[3] Zheng S Fan J Yu F: Viral load dynamics and disease severity in patients infected with SARS-CoV-2 in Zhejiang province, China, January-March 2020: retrospective cohort study. *BMJ.* 2020;369:m1443. 10.1136/bmj.m1443 32317267 PMC7190077

[4] Wang W Xu Y Gao R: Detection of SARS-CoV-2 in Different Types of Clinical Specimens. *JAMA.* 2020;323(18):1843–1844. 10.1001/jama.2020.3786 32159775 PMC7066521

[5] Cheng MP Papenburg J Desjardins M: Diagnostic Testing for Severe Acute Respiratory Syndrome-Related Coronavirus-2: A Narrative Review. *Ann Intern Med.* 2020;172(11):726–734. 10.7326/M20-1301 32282894 PMC7170415

[6] Adams ER Ainsworth M Anand R: Antibody testing for COVID-19: A report from the National COVID Scientific Advisory Panel [version 1; peer review: 1 approved]. *Wellcome Open Res.* 2020;5:139 10.12688/wellcomeopenres.15927.1 PMC7941096 33748431

[7] Pastorino B Touret F Gilles M: Evaluation of heating and chemical protocols for inactivating SARS-CoV-2. *bioRxiv.* 2020; 2020.04.11.036855. 10.1101/2020.04.11.036855 PMC7354533 32521706

[8] Remy MM Alfter M Chiem MN: Effective chemical virus inactivation of patient serum compatible with accurate serodiagnosis of infections. *Clin Microbiol Infect.* 2019;25(7):907.e7–907.e12. 10.1016/j.cmi.2018.10.016 30391583 PMC7128130

[9] Andersson M Barnes E Beneke T: SARS-CoV-2 RNA in blood.2020 10.6084/m9.figshare.12278249.v8

[10] Report of the WHO-China Joint Mission on Coronavirus Disease 2019 (COVID-19).2020 Reference Source

[11] Public Health England 2019-nCoV real-time RT-PCR RdRp gene assay. Report No.: version 1.0 [28-Jan-2020].

[12] Corman VM Landt O Kaiser M: Detection of 2019 novel coronavirus (2019-nCoV) by real-time RT-PCR. *Euro Surveill.* 2020;25(3):2000045. 10.2807/1560-7917.ES.2020.25.3.2000045 31992387 PMC6988269

[13] Zhang W Du RH Li B: Molecular and serological investigation of 2019-nCoV infected patients: implication of multiple shedding routes. *Emerg Microbes Infect.* 2020;9(1):386–389. 10.1080/22221751.2020.1729071 32065057 PMC7048229

[14] Lescure FX Bouadma L Nguyen D: Clinical and virological data of the first cases of COVID-19 in Europe: a case series. *Lancet Infect Dis.* 2020;20(6):697–706. 10.1016/S1473-3099(20)30200-0 32224310 PMC7156120

[15] Duan K Liu B Li C: Effectiveness of convalescent plasma therapy in severe COVID-19 patients. *Proc Natl Acad Sci U S A.* 2020;117(17):9490–9496. 10.1073/pnas.2004168117 32253318 PMC7196837

[16] Chen X Zhao B Qu Y: Detectable serum SARS-CoV-2 viral load (RNAaemia) is closely correlated with drastically elevated interleukin 6 (IL-6) level in critically ill COVID-19 patients. *Clin Infect Dis.* 2020. 10.1093/cid/ciaa449 32301997 PMC7184354

[17] Chen W Lan Y Yuan X: Detectable 2019-nCoV viral RNA in blood is a strong indicator for the further clinical severity. *Emerg Microbes Infect.* 2020;9(1):469–473. 10.1080/22221751.2020.1732837 32102625 PMC7054964

[18] Fang Z Zhang Y Hang C: Comparisons of viral shedding time of SARS-CoV-2 of different samples in ICU and non-ICU patients. *J Infect.* 2020;81(1):147–178. 10.1016/j.jinf.2020.03.013 32209381 PMC7118636

[19] Han MS Seong MW Heo EY: Sequential analysis of viral load in a neonate and her mother infected with SARS-CoV-2. *Clin Infect Dis.* 2020; ciaa447. 10.1093/cid/ciaa447 32297925 PMC7184375

[20] Huang C Wang Y Li X: Clinical features of patients infected with 2019 novel coronavirus in Wuhan, China. *Lancet.* 2020;395(10223):497–506. 10.1016/S0140-6736(20)30183-5 31986264 PMC7159299

[21] Yu F Yan L Wang N: Quantitative Detection and Viral Load Analysis of SARS-CoV-2 in Infected Patients. *Clin Infect Dis.* 2020;ciaa345. 10.1093/cid/ciaa345 32221523 PMC7184442

[22] Young BE Ong SWX Kalimuddin S: Epidemiologic Features and Clinical Course of Patients Infected With SARS-CoV-2 in Singapore. *JAMA.* 2020;323(15):1488–1494. 10.1001/jama.2020.3204 32125362 PMC7054855

[23] Xie C Jiang L Huang G: Comparison of different samples for 2019 novel coronavirus detection by nucleic acid amplification tests. *Int J Infect Dis.* 2020;93:264–267. 10.1016/j.ijid.2020.02.050 32114193 PMC7129110

[24] Wu J Liu J Li S: Detection and analysis of nucleic acid in various biological samples of COVID-19 patients. *Travel Med Infect Dis.* 2020;101673. 10.1016/j.tmaid.2020.101673 32311437 PMC7165102

[25] Cai J Xu J Lin D: A Case Series of children with 2019 novel coronavirus infection: clinical and epidemiological features. *Clin Infect Dis.* 2020;ciaa198. 10.1093/cid/ciaa198 32112072 PMC7108143

[26] Wölfel R Corman VM Guggemos W: Virological assessment of hospitalized patients with COVID-2019. *Nature.* 2020;581(7809):465–469. 10.1038/s41586-020-2196-x 32235945

[27] COVID-19 Investigation Team: Clinical and virologic characteristics of the first 12 patients with coronavirus disease 2019 (COVID-19) in the United States. *Nat Med.* 2020;26(6):861–868. 10.1038/s41591-020-0877-5 32327757 PMC12755114

[28] Peng L Liu J Xu W: SARS-CoV-2 can be detected in urine, blood, anal swabs, and oropharyngeal swabs specimens. *J Med Virol.* 2020. 10.1002/jmv.25936 32330305 PMC7264521

[29] Corman VM Rabenau HF Adams O: SARS-CoV-2 asymptomatic and symptomatic patients and risk for transfusion transmission. *Transfusion.* 2020;60(6):1119–1122. 10.1111/trf.15841 32361996 PMC7267331

[30] Song L Xiao G Zhang X: A case of SARS-CoV-2 carrier for 32 days with several times false negative nucleic acid tests. *medRxiv.* 2020 10.1101/2020.03.31.20045401

[31] Lu R Wang J Li M: SARS-CoV-2 detection using digital PCR for COVID-19 diagnosis, treatment monitoring and criteria for discharge. *medRxiv.* 2020 10.1101/2020.03.24.20042689

[32] Mancuso P Gidaro A Gregato G: Viable circulating endothelial cells and their progenitors are increased in Covid-19 patients. Infectious Diseases (except HIV/AIDS). *medRxiv.* 2020 10.1101/2020.04.29.20085878

[33] Hogan CA Stevens B Sahoo MK: High frequency of SARS-CoV-2 RNAemia and association with severe disease.Infectious Diseases (except HIV/AIDS). *medRxiv.* 2020 10.1101/2020.04.26.20080101 PMC7543277 32965474

[34] Tan W Lu Y Zhang J: Viral Kinetics and Antibody Responses in Patients with COVID-19.Infectious Diseases (except HIV/AIDS). *medRxiv.* 2020 10.1101/2020.03.24.20042382

[35] Chen M Tu C Tan C: Key to successful treatment of COVID-19: accurate identification of severe risks and early intervention of disease progression.Respiratory Medicine. *medRxiv.* 2020 10.1101/2020.04.06.20054890

[36] Bouadma L Wiedemann A Patrier J: Immune alterations during SARS-CoV-2-related acute respiratory distress syndrome.Infectious Diseases (except HIV/AIDS). *medRxiv.* 2020 10.1101/2020.05.01.20087239 PMC7443154 32829467

[37] Ling Y Xu SB Lin YX: Persistence and clearance of viral RNA in 2019 novel coronavirus disease rehabilitation patients. *Chin Med J (Engl).* 2020;133(9):1039–1043. 10.1097/CM9.0000000000000774 32118639 PMC7147278

[38] Chang L Zhao L Gong H: Severe Acute Respiratory Syndrome Coronavirus 2 RNA Detected in Blood Donations. *Emerg Infect Dis.* 2020;26(7):1631–1633. 10.3201/eid2607.200839 32243255 PMC7323524

[39] Ratcliff J Nguyen D Andersson M Simmonds P: Evaluation of Different PCR Assay Formats for Sensitive and Specific Detection of SARS-CoV-2 RNA. *bioRxiv.* 2020 10.1101/2020.06.24.168013

[40] Ratti C Budge G Ward L: Detection and relative quantitation of Soil-borne cereal mosaic virus (SBCMV) and Polymyxa graminis in winter wheat using real-time PCR (TaqMan®). *J Virol Methods.* 2004;122(1):95–103. 10.1016/j.jviromet.2004.08.013 15488626

[41] Niu P Lu R Zhao L: Three Novel Real-Time RT-PCR Assays for Detection of COVID-19 Virus. *China CDC Weekly.* 2020;2(25):453–457. 10.46234/ccdcw2020.116 PMC8393056 34594677

[42] De Madrid AT Porterfield JS: A simple micro-culture method for the study of group B arboviruses. *Bull World Health Organ.* 1969;40(1):113–121. 4183812 PMC2554446

[43] The National SARS-CoV-2 Serology Assay Evaluation Group: Performance characteristics of five immunoassays for SARS-CoV-2: a head-to-head benchmark comparison. *Lancet Infect Dis.* 2020. 10.1016/S1473-3099(20)30634-4 32979318 PMC7511171

[44] Yu X Sun S Shi Y: SARS-CoV-2 viral load in sputum correlates with risk of COVID-19 progression. *Critical care.* 2020;24(1):170. 10.1186/s13054-020-02893-8 32326952 PMC7179376

[45] Liu Y Liao W Wan L: Correlation Between Relative Nasopharyngeal Virus RNA Load and Lymphocyte Count Disease Severity in Patients with COVID-19. *Viral Immunol.* 2020. 10.1089/vim.2020.0062 32297828

[46] Li H Liu L Zhang D: SARS-CoV-2 and viral sepsis: observations and hypotheses. *Lancet.* 2020;395(10235):1517–1520. 10.1016/S0140-6736(20)30920-X 32311318 PMC7164875

[47] Xu Y Li X Zhu B: Characteristics of pediatric SARS-CoV-2 infection and potential evidence for persistent fecal viral shedding. *Nat Med.* 2020;26(4):502–505. 10.1038/s41591-020-0817-4 32284613 PMC7095102

[48] Cheung KS Hung IF Chan PP: Gastrointestinal Manifestations of SARS-CoV-2 Infection and Virus Load in Fecal Samples from the Hong Kong Cohort and Systematic Review and Meta-analysis. *Gastroenterology.* 2020; S0016-5085(20)30448-0. 10.1053/j.gastro.2020.03.065 32251668 PMC7194936

[49] Ng DL Goldgof GM Shy BR: SARS-CoV-2 seroprevalence and neutralizing activity in donor and patient blood. Nat Commun. *Nat Commun.* 2020;11(1):4698. 10.1038/s41467-020-18468-8 32943630 PMC7499171

[50] Luchsinger LL Ransegnola B Jin D: Serological Assays Estimate Highly Variable SARS-CoV-2 Neutralizing Antibody Activity in Recovered COVID19 Patients. *J Clin Microbiol.* 2020;11(1):JCM.02005–20. 10.1128/JCM.02005-20 32917729 PMC7685895

[51] Allison KM Faddy HM Margaritis A: The impact on blood donor screening for human immunodeficiency virus, hepatitis C virus, and hepatitis B virus using plasma from frozen-thawed plasma preparation tubes. *Transfusion.* 2016;56(2):449–456. 10.1111/trf.13372 26456378

[52] McLeish NJ Witteveldt J Clasper L: Development and assay of RNA transcripts of enterovirus species A to D, rhinovirus species a to C, and human parechovirus: assessment of assay sensitivity and specificity of real-time screening and typing methods. *J Clin Microbiol.* 2012;50(9):2910–2917. 10.1128/JCM.01172-12 22740708 PMC3421820

[53] Anwar A Wan G Chua KB: Evaluation of pre-analytical variables in the quantification of dengue virus by real-time polymerase chain reaction. *J Mol Diagn.* 2009;11(6):537–542. 10.2353/jmoldx.2009.080164 19815693 PMC2765752

[54] Public Health England: COVID-19: safe handling and processing for samples in laboratories.In: GOV.UK. GOV.UK.2020; [cited 5 May 2020]. Reference Source

[55] CDC: Coronavirus Disease 2019 (COVID-19).In: *Centers for Disease Control and Prevention* 2020 Reference Source

[56] Laboratory biosafety guidance related to coronavirus disease (COVID-19). [cited 14 May 2020]. Reference Source

