# Supplementary material for: SARS-CoV-2 RNA detected in blood products from patients with COVID-19 is not associated with infectious virus
Source: Wellcome Open Res. 2020 Oct 12;5:181. Originally published 2020 Jul 29. [Version 2] doi: 10.12688/wellcomeopenres.16002.2 (PMC7689603; doi:10.12688/wellcomeopenres.16002.2)
Supplement: Peer review history

# Peer review: Reviewer response for version 2

**DOI:** 10.21956/wellcomeopenres.17977.r41565

**Published:** 2020-11-25

**Authors:** Le Sun

**Affiliations:** 1 AnyGo Technology, Beijing, China; 2 AbMax Biotechnology, Beijing, China

Since SARS-CoV-2 is a RNA virus, the sample pre-treatment is very critical to the RNA stability and virus viability. So 1) please give the details of the serum preparation protocol including the types of tubes, the duration prior to serum preparation, any heat-inactivation, et al. 2) should try to spike the RNA standards into normal human whole blood to assess the damage of serum preparation to RNA if possible. 3) Please state what is the SARS-CoV-2 antigen that was used for Siemen's antibody detection kit. Correlations between neutralizing activities with antibodies titers to N protein, RBD, S1, and S will be very different.

Is the work clearly and accurately presented and does it cite the current literature?

Yes

If applicable, is the statistical analysis and its interpretation appropriate?

Yes

Are all the source data underlying the results available to ensure full reproducibility?

Yes

Is the study design appropriate and is the work technically sound?

Yes

Are the conclusions drawn adequately supported by the results?

Yes

Are sufficient details of methods and analysis provided to allow replication by others?

Yes

Reviewer Expertise:

antibody drug, COVID-19 vaccine development and diagnostic  tools for cancers and infectious diseases, contract antibody and assay development.

I confirm that I have read this submission and believe that I have an appropriate level of expertise to confirm that it is of an acceptable scientific standard.

# Peer review: Reviewer response for version 2

**DOI:** 10.21956/wellcomeopenres.17977.r40840

**Published:** 2020-10-15

**Authors:** William L. Irving

**Affiliations:** 1 NIHR Nottingham Biomedical Research Centre, Nottingham University Hospitals NHS Trust, Nottingham, UK

Thank you for the update on this article. I am impressed that the team have been able to conduct additional studies on the relationship between antibody status and RNA detection, which I think adds to the value of the article.

 I have no further comments.

Is the work clearly and accurately presented and does it cite the current literature?

Yes

If applicable, is the statistical analysis and its interpretation appropriate?

Yes

Are all the source data underlying the results available to ensure full reproducibility?

Yes

Is the study design appropriate and is the work technically sound?

Yes

Are the conclusions drawn adequately supported by the results?

Yes

Are sufficient details of methods and analysis provided to allow replication by others?

Yes

Reviewer Expertise:

Clinical virology; pathogenesis, epidemiology and treatment of viral hepatitis

I confirm that I have read this submission and believe that I have an appropriate level of expertise to confirm that it is of an acceptable scientific standard.

# Peer review: Reviewer response for version 1

**DOI:** 10.21956/wellcomeopenres.17551.r39777

**Published:** 2020-08-24

**Authors:** William L. Irving

**Affiliations:** 1 NIHR Nottingham Biomedical Research Centre, Nottingham University Hospitals NHS Trust, Nottingham, UK

- This work sets out to determine the frequency and significance of detection of SARS-CoV2 RNA in blood. This is a pertinent and relevant question to address. The methods are well described in great detail.
- The literature review yielded a point estimate for the prevalence of vRNA in blood products in the 28 days following symptomatic infection of 10% (95%CI 5-18%). Copy number in positive samples was uniformly low. When testing local samples, 20/167 (12%) patients had RT-PCR positive samples at any time point tested. vRNAaemia levels tended to decrease over time since onset of symptoms/diagnosis, and also correlated with critical disease severity.
- Attempts to culture virus from RT-PCR positive samples were unsuccessful, suggesting the virus was not replication competent.
- In the discussion, the sentence “Different clinical and laboratory infection control practices to be considered for specific sample types, to determine the frequency and duration of carriage and to assess whether infectious virus can be detected.” isn’t really a sentence.
- This is an excellent study providing valuable data which will contribute to our understanding of the significance of detection of RNA-aemia.
- My one substantive comment relates to possible neutralisation of virus present in the bloodstream by antibodies. I don’t think (apologies if I’ve missed it) this is mentioned in the discussion. There is considerable patient-to-patient variation in generation of Nabs. It would be of great interest to know the frequency and titre of neutralising antibody in each of the sera tested. This would allow (i) assessment of whether detectable RNA correlates (most likely inversely) with the presence/titre of Nab, and (ii) the selection of RNA positive sera with the lowest Nab titre for virus culture, to address the hypothesis that failure to culture virus may be linked to the presence of high titres of Nab. If it is not practicable to do this, then I think it would be worth adding a sentence or two to the discussion to raise this possibility.

Is the work clearly and accurately presented and does it cite the current literature?

Yes

If applicable, is the statistical analysis and its interpretation appropriate?

Yes

Are all the source data underlying the results available to ensure full reproducibility?

Yes

Is the study design appropriate and is the work technically sound?

Yes

Are the conclusions drawn adequately supported by the results?

Yes

Are sufficient details of methods and analysis provided to allow replication by others?

Yes

Reviewer Expertise:

Clinical virology; pathogenesis, epidemiology and treatment of viral hepatitis

I confirm that I have read this submission and believe that I have an appropriate level of expertise to confirm that it is of an acceptable scientific standard, however I have significant reservations, as outlined above.

# Response

**Published:** 2020-09-29

**Authors:** Philippa Matthews

**Affiliations:** Nuffield Department of Medicine, Peter Medawar Building for Pathogen Research, University of Oxford, UK

Thank you to the reviewer for the helpful feedback and positive comments.

With regard to the error in the discussion, we have corrected the sentence that contained grammatical errors to read as follows: ‘Different clinical and laboratory infection control practices should be considered for specific sample types, ideally based on an understanding of the frequency and duration of carriage and assessment of whether infectious virus can be detected’.

The question about antibody detection in our sample set is a very good one. We have been able to add data for antibody titres in a subset of our samples (n=160), determined by the Siemens immunoassay, a validated commercial platform. We have added the raw data to an updated supplementary metadata table on-line, and have generated a new figure presenting these results (Fig 5). Our analysis shows that, as expected, the samples that are PCR-positive were significantly less likely to be antibody positive (p=0.019; panel A). However, there was no significant difference in quantitative antibody titres between PCR-positive and negative samples (p=0.2, panel B). Presenting the antibody titres in PCR-positive sera shows no clear cut-off in IgG titre that is associated with PCR-positivity (panel C), and demonstrates that positive antibody status was not pre-requisite to preventing CPE in vitro. We have added to the discussion to point out that we cannot determine a causal relationship between the development of antibody and loss of detectable RNA in samples, particularly as we have not specifically measured neutralising activity in this sample set, but there are emerging data from other sources to support a correlation between titres and *in vitro* neutralisation. We have modified the methods section to incorporate description of the Siemens assay, updated the results to present these data, and added a short paragraph to the discussion to provide context and interpretation. The antibody data are now also referenced in the results section of the abstract.
